# Characterization and phylogenetic epitope mapping of CD38 ADPR cyclase in the cynomolgus macaque

**DOI:** 10.1186/1471-2172-5-21

**Published:** 2004-09-21

**Authors:** Enza Ferrero, Monia Orciani, Paola Vacca, Erika Ortolan, Sergio Crovella, Fausto Titti, Franca Saccucci, Fabio Malavasi

**Affiliations:** 1 Department of Genetics, Biology & Biochemistry, University of Torino, Via Santena 19 and the CeRMS Research Center for Experimental Medicine, 10126 Torino, Italy; 2 Institute of Biology and Genetics, Marche Polytechnic University, Via Ranieri 69, 60131 Ancona, Italy; 3 Department of Reproductive and Developmental Sciences, University of Trieste, Via dell'Istria 65/1, 34137 Trieste, Italy; 4 Department of Parasitic, Infectious and Immune-mediated Diseases, Istituto Superiore di Sanità, Viale Regina Elena 299, 00161 Rome, Italy

## Abstract

**Background:**

The CD38 transmembrane glycoprotein is an ADP-ribosyl cyclase that moonlights as a receptor in cells of the immune system. Both functions are independently implicated in numerous areas related to human health. This study originated from an inherent interest in studying CD38 in the cynomolgus monkey (*Macaca fascicularis*), a species closely related to humans that also represents a cogent animal model for the biomedical analysis of CD38.

**Results:**

A cDNA was isolated from cynomolgus macaque peripheral blood leukocytes and is predicted to encode a type II membrane protein of 301 amino acids with 92% identity to human CD38. Both RT-PCR-mediated cDNA cloning and genomic DNA PCR surveying were possible with heterologous human *CD38 *primers, demonstrating the striking conservation of *CD38 *in these primates. Transfection of the cDNA coincided with: (i) surface expression of cynomolgus macaque CD38 by immunofluorescence; (ii) detection of ~42 and 84 kDa proteins by Western blot and (iii) the appearance of ecto-enzymatic activity. Monoclonal antibodies were raised against the cynomolgus CD38 ectodomain and were either species-specific or cross-reactive with human CD38, in which case they were directed against a common disulfide-requiring conformational epitope that was mapped to the C-terminal disulfide loop.

**Conclusion:**

This multi-faceted characterization of CD38 from cynomolgus macaque demonstrates its high genetic and biochemical similarities with human CD38 while the immunological comparison adds new insights into the dominant epitopes of the primate CD38 ectodomain. These results open new prospects for the biomedical and pharmacological investigations of this receptor-enzyme.

## Background

Just over a decade after being identified as a leukocyte surface antigen with receptorial activity [1,2], CD38 was re-classified among the ADP-ribosyl (ADPR) cyclases [3,4]. These are a group of related membrane-bound or soluble enzymes, comprising CD157 and *Aplysia *ADPR cyclase [5,6], which have the unique capacity to convert NAD to cyclic ADP ribose (cADPR) or nicotinic acid-adenine dinucleotide phosphate (NAADP), part of a new generation of endogenous activators of intracellular Ca^2+ ^release [6].

Human CD38 is a broadly expressed type II transmembrane glycoprotein of ~45 kDa in its monomeric form [7]. This consists of a short intracytoplasmic (IC) tail, a transmembrane domain and a major extracellular domain (ECD) formed by 256 of the 300 constituent amino acids of the CD38 polypeptide [7]. Homodimeric and homotetrameric forms have also been described [8,9] and a 3-D dimer structure obtained by homology modeling to *Aplysia *cyclase [10]. The CD38 ECD, where both receptor and enzymatic activities reside, harbours a 12 cysteine/6 disulfide signature common to the members of this family. According to a growing body of experimental evidence, the disulfides mediate control of the ECD conformation and function since reduction modifies CD38 enzymatic activity and homodimerization [11,12], and sensitivity to proteolysis and monoclonal antibody (mAb) binding [13].

The mobilization of intracellular Ca^2+ ^caused by the CD38/cADPR/NAADP axis has been implicated in a variety of physiological and pathological processes including insulin secretion and diabetes [14], myometrial contractility and pregnancy [15], airway smooth muscle contractility and hyperreactivity [16], vascular smooth muscle contraction [17], osteoclast activity [18], and the functions of the immune [19], renal [20] and exocrine gland [21] systems. The assortment of effects caused by CD38 ligation and transmembrane signalling is also broad though mostly described in hematopoietic cells, and ranges from lymphocyte proliferation and cytokine release [2,22-24], regulation of B and myeloid cell development and survival [25-28], inhibition of human immunodeficiency virus (HIV) entry [29], to induction of dendritic cell maturation [30]. In addition, ligation of human pancreatic islet cells by anti-CD38 autoantibodies induces insulin release [31]. CD38 is also a clinically useful marker of HIV infection progression [32] and therapy-requiring B-CLL [33].

In this study, we describe the molecular cloning and functional expression of CD38 from the cynomolgus macaque. In addition, with a panel of newly-raised mAbs, we comparatively analyse the macaque and human CD38 ECDs and identify new structural-functional characteristics of CD38 epitopes.

## Results

### Cloning CD38 cDNA from cynomolgus macaque

**Figure 1:**
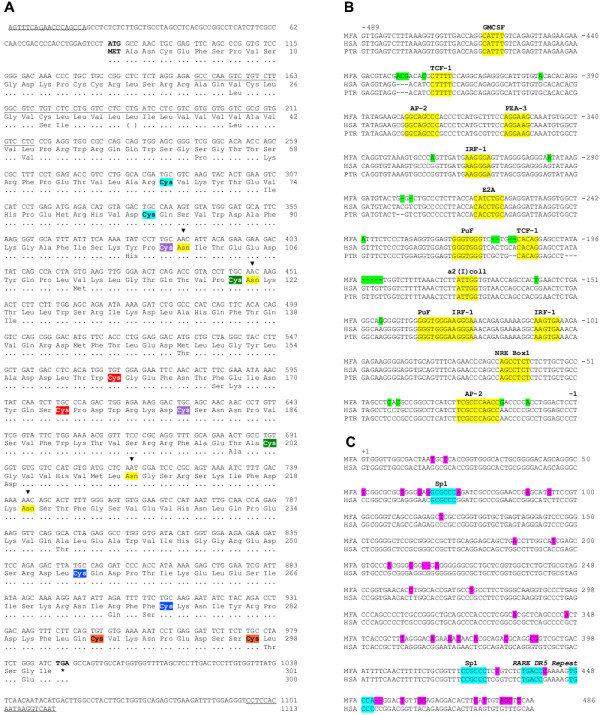
**Cynomologus macaque CD38 cDNA, promoter and 5' end of intron 1. **A. Primers used to clone the cDNA are wavy-underlined. Members of the 12-cysteine array of the ectodomain are color-boxed; cysteines that pair in disulfide formation are boxed in the same color. Differences in the human CD38 amino acid sequence are indicated under the macaque sequence. The transmembrane domain is underlined and glycosylation sites indicated. The sequence accession number is AY555148. B. Nucleotide sequence of the CD38 promoter from *Macaca fascicularis *(MFA) (Acc. No. AY622999), *Homo sapiens *(HSA) (Acc. No. AF001985) and *Pan troglodytes *(PTA) (Acc. No. AY623001). The nucleotide sequences begin at -1 immediately upstream of the ATG initiation codon. Potential transcription regulatory motifs common to the 3 species, identified with TRANSFAC^® ^[62], are boxed and the relevant transcription factor indicated above. C. 5' end of intron 1 of macaque and human *CD38*, Acc. No. AY623000 and AF088883, respectively.

Activation of human peripheral blood mononuclear cells (PBMC) with phytohemagglutinin (PHA) strongly upregulates expression of CD38 in human T lymphocytes [34]. Therefore, to isolate a CD38 cDNA, PHA-activated cynomolgus PBMC were chosen as the source of RNA for amplification by RT-PCR using primers derived from the human *CD38 *5' and 3' untranslated regions. The 1113 base-pair (bp) insert contained an open reading frame of 906 bp (Figure 1A) that was 95% identical to the human CD38 sequence. The cDNA encodes a 301 amino acid (aa) polypeptide with the typical CD38 type II membrane protein structure, *i.e*., a short cytosolic tail (residues 1–21), a transmembrane region (residues 22–44), and an ECD (residues 45–301) containing the signature 12-cysteine array. Alignment of the macaque and human CD38 polypeptides showed 92% identity and 94% similarity. There is complete conservation of the IC region while there are five conservative changes in the transmembrane region where macaque CD38 has one more residue than human CD38. Macaque CD38 has four potential *N*-linked glycosylation sites, as in human CD38; three are co-linear. Furthermore, macaque CD38 shows conservation of the 4 acidic residues (Glu^148^, Asp^149^, Asp^157^, Glu^228^) and 2 tryptophans (Trp^127 ^and Trp^191^) that play a critical role in the ADP-ribosyl cyclase/cADPR hydrolase activities of human CD38 [35]. Lys^130 ^is also maintained suggesting that, like human CD38, binding of ATP to this residue may lead to inhibition of the hydrolase activity [36]. Likewise, macaque CD38 conserves Arg^271 ^which is ADP-ribosylated in human CD38, causing inactivation [37].

### Common genomic organization and regulatory features of macaque and human CD38 genes

Human *CD38 *has been characterized as a single-copy, 8-exon gene that spans ~70 kb [5,38] and not only CD38 but ADPR cyclase genes in general are highly conserved from mollusks to humans [39,40]. In addition, the Southern blot hybridization patterns of macaque and human genomic DNAs digested with *Eco*RI, *Bam*HI and *Hind*III and probed with their homologous CD38 cDNA are similar (*data not shown*), indicating that the structural organization of macaque and human *CD38 *is highly conserved. This was further demonstrated by the finding that the same primers previously used to amplify the 8 human *CD38 *exons [41] also amplified 8 *CD38 *exons from macaque genomic DNA. The putative macaque exons could be perfectly aligned with their human counterparts [5] in number, size and splice site of their exons (Table 1). All intron-exon boundaries conformed to the GT-AG rule, most 5' splice donor and 3' splice acceptor sequences of the 7 introns were identical.

**Table 1:** Exons and introns of cynomolgus macaque *CD38*

Exon	5' splice donor	Intron	3' splice acceptor	Exon
1	ATGAGgtggg	I	cacagACATG	2
	MetAr^79^		gHisV	
2	ACAGGgtaat	II	cttagACTCT	3
	snLys^122^		ThrLe	
3	TTTCGgtgag	III	tttagAAATA	4
	rPheG^168^		luIle	
4	GCAGGgtaag	IV	ttaagTTTGC	5
	rgArg^196^		PheAl	
5	AACAGgtaac	V	tttagCACTT	6
	AsnSe^221^		rThrP	
6	TCCAGgtata	VI	cccagAGACT	7
	SerAr^251^		gAspL	
7	TACAGgtaat	VII	cacagACCTG	8
	TyrAr^280^		gProA	

Transcription of TATA-boxless human *CD38 *initiates at multiple start sites [5,38] while induction of gene expression by retinoids is controlled by a retinoic acid responsive element (RARE) at the beginning of intron 1 [42]. To identify conserved *cis*-regulatory sequences, ~500 bp upstream of the initiation codon ATG were amplified by PCR from genomic DNA of cynomolgus macaque but also from chimpanzee (*Pan troglodytes*), to strengthen the alignment with the human *CD38 *promoter sequence, and numerous conserved general and immune-related potential binding sites were found (Figure 1B). The alignment of the 5' end of intron 1 from macaque and human CD38 shows the presence of the RARE (Figure 1C), suggesting that this and perhaps other molecular mechanisms involved in regulating *CD38 *are conserved between these primates.

### Expression of macaque CD38 reveals an active cyclase of ~42 kDa

To analyse surface expression and function of macaque CD38, the cDNA insert was subcloned into the pcDNA3.1 expression vector and a similar construct was prepared containing the human CD38 cDNA (*see Materials & Methods*). DNAs were transfected for heterologous expression in the NIH/3T3 cell line which does not express CD38 [43]. Given that cross-reactivity of OKT10 anti-human CD38 mAb has been exploited to detect CD38 in rhesus macaque hematopoietic cells [44,45], clones expressing cynomolgus CD38 were identified by indirect immunofluorescence (IF) with OKT10 (see below). The stable cell line expressing macaque CD38 was designated NIH/mac38, while NIH/hum38 is the human CD38-expressing cell line.

**Figure 2:**
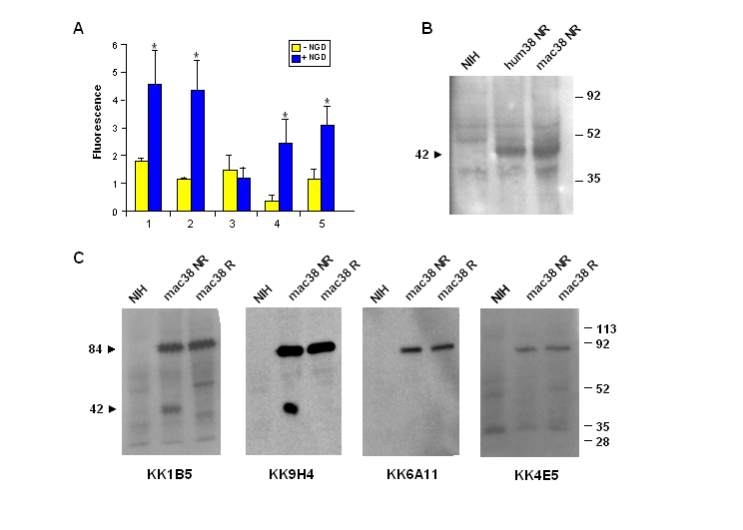
**Enzymatic activity and western blot analysis of cynomolgus CD38. **A. Ecto-cyclase activity was evaluated by measuring conversion of NGD to fluorescent cGDPR by NIH/mac38 (1), NIH/hum38 (2), NIH/3T3 (3), or cynomolgus macaque RBCs (4) and human RBCs (5). Each bar represents mean ± SD; (*) means significantly higher than controls (*P *< 0.05, *t*-test, *n *= 3 experiments). Y axis = fluorescence emission 410 nm. B. Western blot of lysates from parental NIH/3T3 cells (NIH), NIH/hum38 (hum38) and NIH/mac38 cells (mac38) analysed by 10% SDS-PAGE under non-reducing (NR) conditions. Blots were probed with AT1 anti-human CD38 mAb. C. Western blot of parental NIH/3T3 and NIH/mac38 cell lysates analysed by 8% SDS-PAGE in non-reducing (NR) or reducing (R) conditions and probed with the indicated anti-cynomolgus CD38 mAbs. In B and C, molecular weight markers (in kDa) are indicated on the right.

The ecto-cyclase activity of macaque CD38 was evaluated by incubating the NIH/mac38 clone with nicotinamide guanine dinucleotide (NGD), an NAD analog which is converted by ADP-ribosyl cyclases such as CD38 to cyclic GDP ribose (cGDPR). Unlike cADPR, cGDPR is a stable, fluorescent end-product which can be detected in cell supernatants [46]. Increased fluorescence following incubation with NGD was detected in supernatants of macaque and human CD38 transfectants, demonstrating that macaque CD38 is enzymatically active (Figure 2A).

In human red blood cells (RBCs), CD38 is the only source of ecto-cyclase activity [47] which was also found on the surface of cynomolgus macaque RBCs (Figure 2A), suggesting a further parallel with human CD38.

To establish the approximate molecular weight of macaque CD38, SDS-PAGE and Western blot analysis were performed with lysates prepared from NIH/mac38 and NIH/hum38 cells. Blots were probed with the AT1 anti-human CD38 mAb which detected a band of ~42 kDa in both transfectants (Figure 2B).

### Production of anti-macaque CD38 mAbs

**Figure 3:**
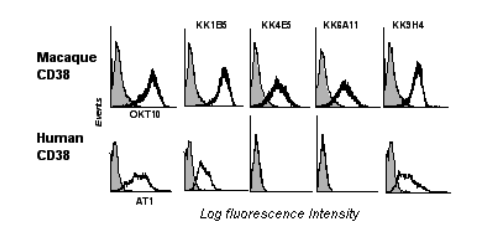
**Reactivity of anti-cynomolgus CD38 mAbs. **Top row: Flow cytometric profiles obtained by IF of parental NIH/3T3 (grey profile) and NIH/mac38 expressing cynomolgus CD38 (white profile) with OKT10 anti-human CD38 mAb and anti-cynomolgus CD38 mAb panel. Profiles obtained by staining both cell lines with CBT3G IgG control mAb completely overlap with the grey profile. Bottom row: Flow cytometric profiles obtained with NIH/hum38 and the indicated mAbs (white profile). Grey profile is the reactivity of CBT3G.

To raise mouse mAbs against macaque CD38, live NIH/mac38 cells were used for immunization. Four mAbs, KK1B5 (IgG1), KK4E5 (IgG2a), KK6A11 (IgG2a) and KK9H4 (IgG1) were selected for further analyses. All four anti-macaque CD38 mAbs reacted by IF with NIH/mac38 but were negative with the parental cell line (Figure 3). Only two mAbs (KK1B5 and KK9H4) reacted with NIH/hum38.

The anti-macaque CD38 mAbs were further assessed by IF for binding to cynomolgus PBMC and to SL-691 and SL-999, two cynomolgus macaque B lymphoblastoid cell lines. All four mAbs reacted with PBMC from cynomolgus macaques, indicating they recognize native cynomolgus CD38, and they strongly stained cells of the two B cell lines (Table 2).

**Table 2:** Reactivity of anti-macaque CD38 mAbs

	**Monoclonal antibodies**				
**Cell lines**	**KK1B5**	**KK4E5**	**KK6A11**	**KK9H4**	**Control**
**Macaque CD38^+^**					
NIH/mac38	**+++^*a*^**	**+++**	**++**	**+++**	**-**
SL-691	**++**	**++**	**++**	**++**	**-**
SL-999	**++**	**++**	**++**	**++**	**-**
PBMC	**++**	**++**	**++**	**++**	**-**
**Human CD38^+^**					
NIH/hum38	**++**	**-**	**-**	**++**	**-**
RAJI	**++**	**-**	**-**	**++**	**-**
Jurkat	**++**	**-**	**-**	**++**	**-**
PBMC	**++**	**-**	**-**	+	**-**
**Control**					
NIH/3T3	**-**	**-**	**-**	**-**	**-**

^*a*+++, very strong reactivity; ++, strong reactivity; +, weak reactivity; -, no reactivity.^

Additional Western blot analyses were carried out with the anti-macaque CD38 mAbs. MAbs KK1B5 and KK9H4 confirmed detection of a ~42 kDa band in NIH/mac38 cell lysates but also recognized a second band of ~84 kDa. (Figure 2C). The doublet was also identified in SL-999 cynomolgus B cells (*data not shown*). Instead mAbs KK4E5 and KK6A11 only recognized a band of ~84 kDa, even in reducing conditions. No bands were detected by these mAbs in parental NIH/3T3 cells.

### Anti-macaque CD38 mAbs recognize either species-specific/DTT-resistant epitopes or a human cross-reactive/DTT-sensitive epitope

**Figure 4:**
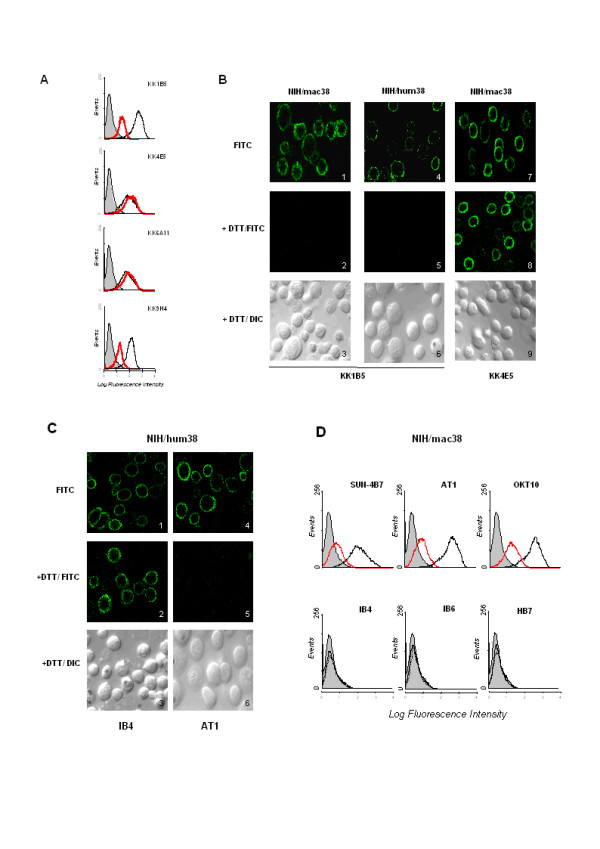
**Reactivity of anti-macaque CD38 mAbs with native and DTT-treated NIH/macCD38 and NIH/humCD38. **A. Flow cytometric profile of NIH/mac38 cells treated for 45 min at 37°C with 10 mM DTT (red profile) or without DTT (black profile) and then tested for binding of anti-cynomolgus CD38 mAbs by IF. Grey profile shows reactivity of CBT3G mAb (isotype control). B. Results of IF/DTT experiments illustrated by confocal microscopy. Transfectants are indicated at the top of the panel. Left vertical triplet of images (from top to bottom): (1) reactivity of mAb KK1B5 with native cynomolgus CD38 cells stained with FITC; (2) reactivity with DTT-treated cells stained by FITC; (3) cells in plate 2 viewed by differential interference contrast (DIC). (4–6): *idem *mAb KK1B5 with NIH/humCD38; (7–9): *idem *mAb KK4E5 mAb with NIH/macCD38. C. IF/confocal microscopy of NIH/humCD38, with/without DTT. Left vertical triplet of images (from top to bottom) shows staining with mAb IB4 and FITC of control (1), DTT-treated visualized by FITC (2) and DIC (3). Right trio: results with mAb AT1 (4–6). D. Flow cytometric profiles of control (black profile) and DTT-treated (red profile) NIH/macCD38 binding by anti-human CD38 mAbs and FITC. Grey profile shows reactivity of CBT3G mAb (isotype control).

The observation that mAbs KK4E5 and KK6A11 recognize only cynomolgus CD38 while mAbs KK1B5 and KK9H4 also recognize human CD38 indicates that the two mAb subsets are directed towards different epitopes. To evaluate the contribution of disulfides to these epitopes, mAb reactivity was assessed after treating NIH/mac38 with dithiothreitol (DTT). Reduction had no effect on binding of the species-specific mAbs (KK4E5 and KK6A11) but significantly reduced binding of mAbs KK1B5 and KK9H4 (Figure 4A), indicating that the latter recognize a disulfide-requiring conformational epitope of the cynomolgus CD38 ECD, and predicting they should recognize a similar epitope in human CD38. The results (Figure 4B) confirm that treatment of NIH/hum38 with DTT decreased binding of mAbs KK1B5 and KK9H4, indicating that cynomolgus macaque and human CD38 have a conformational epitope in common.

### Anti-human CD38 mAbs are also species-specific/DTT-resistant or cross-reactive/DTT-sensitive

Reciprocal experiments were performed to see if the correlation between cross-reactivity and epitope sensitivity to DTT was also valid for a panel of 6 well-known mAbs raised against human CD38. MAbs IB4, IB6, OKT10, SUN-4B7, AT1 and HB7 were assessed for binding to native and DTT-treated NIH/hum38, and for cross-reactivity with cynomolgus CD38. Binding of mAbs IB4, IB6 and HB7 to human CD38 was unaffected by DTT and none bound cynomolgus CD38 (Figure 4C and 4D). On the contrary, binding of mAbs OKT10, SUN-4B7 and AT1 to human CD38 was significantly reduced by DTT and all three mAbs bound cynomolgus CD38 (Figure 4D) in a DTT-sensitive manner.

### The epitope recognized by cross-reactive cynomolgus anti-CD38 mAbs maps to the C-terminal disulfide loop

The observation that mAbs KK1B5 and KK9H4 (raised against cynomolgus CD38) and mAbs OKT10, AT1 and SUN-4B7 (raised against human CD38) all bind native but not reduced cynomolgus and human CD38 is compatible with their binding the same epitope. *A priori *knowledge of the human CD38 epitope map previously established that OKT10 binding is abrogated by deletion of one or both of the C-terminal Cys residues (Cys^287 ^and Cys^296^) predicted to pair in disulfide bond formation [48] and that mAbs OKT10, AT1 and SUN-4B7 mutually compete for binding to the human CD38 ECD [49]. This would position the common epitope of cynomolgus and human CD38 in the C-terminal disulfide loop formed by Cys^288 ^and Cys^297 ^in cynomolgus CD38 (Cys^287^/Cys^296 ^in human CD38).

**Figure 5:**
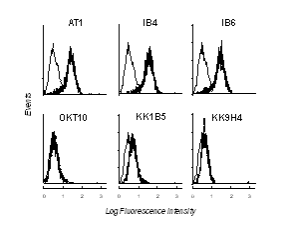
**Reactivity of macaque/human CD38 cross-reactive mAbs with MT2^Δ285 ^cells expressing truncated human CD38. **Flow cytometric profiles obtained by IF with the indicated mAbs and MT2 T lymphoid cells stably expressing human CD38 deleted of the 15 C-terminal residues (heavy black line profile). Isotype control (light black line profile).

**Figure 6:**
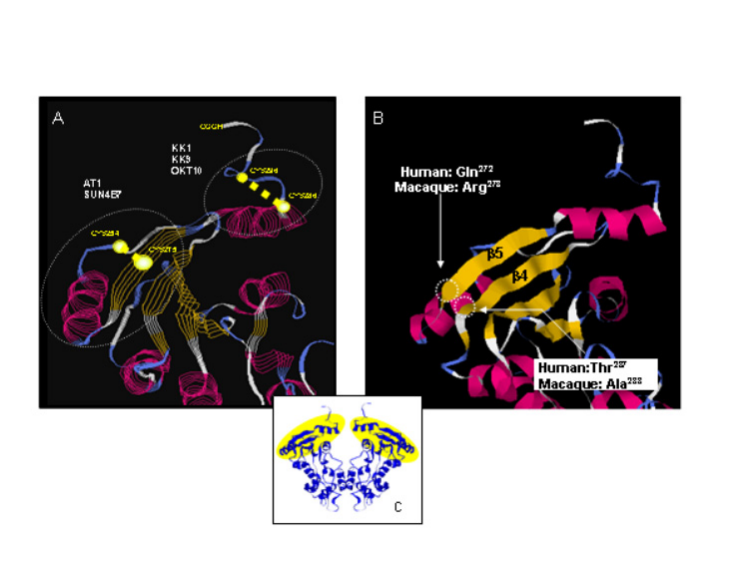
**Molecular model of human CD38 epitope map. **A. Homology model of human CD38 derived from *Aplysia *ADPR cyclase 3-D model made with RasWin Molecular Graphics showing footprints of the relevant anti-macaque and anti-human CD38 mAbs. Close-up of two C-terminal disulfide loops delimited by Cys^254^-Cys^275^, and Cys^285^-Cys^296^. Position of cysteine residues and disulfide bonds are indicated in yellow. Sequence is represented by secondary structure (red, alpha helix; blue, beta strand; white, turn). B. Same model illustrating the two beta strands implicated in binding of human species-specific mAbs and the residue changes in macaque CD38 that may account for their lack of cross-reactivity. C. Model of the dimeric form of the human CD38 ECD showing where epitopes are located.

To test this possibility, we analysed reactivity of these mAbs with the CD38-negative MT2 human T cell line stably transfected with CD38^Δ285^, a human CD38 deletion mutant which lacks the 15 C-terminal amino acids and loses OKT10 binding [29]. IF analysis demonstrates that the 15 C-terminal amino acids of human CD38 are required for binding of mAbs KK1B5, KK9H4 and OKT10 (Figure 5). In contrast, mAbs SUN-4B7 and AT1 maintained binding to MT2/CD38^Δ285 ^(*data not shown*). These data are consistent with the presence of two close conformational epitopes in human and cynomolgus CD38: one identified by mAbs KK1B5, KK9H4 and OKT10 located in the last (6^th^) disulfide loop, the other by mAbs SUN-4B7 and AT1 mapping to the penultimate (5^th^) C-terminal disulfide loop involving Cys^254^-Cys^275 ^(Figure 6). Note that human-cynomolgus CD38 amino acid sequence identity in the 5^th ^loop is 20/22 amino acids, and 10/10 amino acids in the 6^th ^loop.

## Discussion

In this study, we describe the molecular cloning and functional expression of the CD38 receptor/enzyme from the cynomolgus macaque. The cDNA described here presents the expected homology to human CD38 considering that the macaque-human lineages diverged some 25 million years ago [50] and their genomes are 93–95% identical. This homology was exploited in RT-PCR cloning and in our genomic PCR survey of cynomolgus *CD38*. Indeed, human *CD38 *primers proved to be equally agile with *CD38 *in other members of the Primate order such as the chimpanzee, the gibbon (*Hylobates concolor*) and the rhesus monkey (*Macaca mulatta*) (MO, FS and SC, unpublished observations).

The conceptual translation of the cynomolgus macaque CD38 cDNA yielded a polypeptide with the characteristic type II structure, size, catalytic core residues and 12-cysteine ECD array common to CD38 orthologs. With respect to human CD38, cynomolgus CD38 has an extra residue in the transmembrane domain but no difference was found in the IC tail, where human CD38 is reported to interact with the SH2 domain of Lck [51] in lipid rafts [52].

Cross-reactivity of anti-human CD38 mAbs was exploited in the initial part of the protein analyses although these give discrepant results in binding to leukocytes from other primates. For example, OKT10, but not Leu17 or T16, binds bone marrow from rhesus macaques [44] whereas HIT2 stains horse lymphocytes but not leukocytes from baboon, cynomolgus macaque, rhesus macaque, pig, sheep, cow, dog, cat or rabbit [53]. In addition, the behaviour of cross-reactive mAbs may not be reproducible in another species as illustrated by anti-human CD34 mAbs: 6 out of 13 mAbs cross-reacted with cynomolgus bone marrow but only 3 of these correctly identified the functional cynomolgus equivalent of the human progenitor cell in clonogenic assays [54]. To avoid similar pitfalls, we raised mAbs to macaque CD38.

The apparent molecular weight of macaque and human CD38 were indistinguishable by SDS-PAGE. The macaque polypeptide weighs 34.4 kDa and has four *N*-linked glycosylation motifs, suggesting it is probably glycosylated, like human CD38 [55], to give rise to the 42 kDa band. We wanted to confirm this result with anti-cynomolgus CD38 mAbs but when lysates were probed with mAbs KK1B5 and KK9H4, the 42 kDa band was always accompanied by an 84 kDa band, and the doublet was also observed with lysates obtained from SL-999 macaque B cells expressing CD38 in its native milieu. The KK4E5 and KK6A11 mAbs instead detected only the 84 kDa band in transfectants, and the band was unaffected by addition of DTT. CD38 dimers and tetramers have been abundantly reported and postulated to be formed by diverse mechanisms such as intermolecular disulfides [12,51], non-covalent association [56] and transglutamination [9]. Therefore, our interpretation of the upper band is that it represents a CD38 dimer, possibly a non-covalently associated form compatible with lysate preparation in NP-40 detergent which stabilizes such dimers [56], or a transglutaminase-linked form. Although the data are consistent with KK4E5 and KK6A11 recognizing a unique epitope in macaque CD38 dimers, it is also possible that these mAbs have another unknown specificity and that further experiments are needed to fully characterize their specificity.

The availability of the macaque CD38 amino acid sequence and its alignment with the human homolog gave a new dimension to the analysis of mAb cross-reactivity and ultimately led to a better understanding of CD38 epitopes. Macaque and human CD38 are strikingly conserved yet only two of the four anti-macaque CD38 mAbs cross-reacted, as did only three of the six anti-human CD38 mAbs in reciprocal experiments. The key finding was that a mAb's capacity to cross-react always correlated with the sensitivity of its target to reduction which, added to the prior knowledge of the human CD38 epitope map, crystal structure and active site [57], allowed us to footprint the binding sites of cross-reactive anti-CD38 mAbs.

The classification of anti-primate CD38 mAbs as species-specific/DTT-resistant (type I) or cross-reactive/DTT-sensitive (type II) and directed against a conformational epitope may be of practical importance. Firstly, our results demonstrate the necessity for careful antibody selection when performing biological assays involving detection of CD38 expression in circumstances of cell membrane redox perturbation, *e.g*., detection of membrane CD38 in apoptotic cells might be positive according to type I mAbs and negative by type II (Alla Egorova, personal communication). Secondly, simultaneous use of the two types of mAbs can provide information about CD38 expression (type I) while the type II mAb can give indications on its conformation. Thirdly, it is possible that potent Ca^2+^-mobilizing agonistic mAbs are more likely to be type I mAbs since this subgroup includes IB4, which is the only anti-human CD38 mAb to mobilize Ca^2+^, and NIM-R5, a rat anti-murine CD38 mAb [58] which also mobilizes Ca^2+ ^and whose binding to murine CD38 transfectants was not affected by DTT (EF, personal communication). Finally, autoantibodies to CD38 have been detected in diabetes and thyroiditis and it would be interesting to identify the epitopic culprits.

## Conclusions

Some of the essential biological features of CD38 in *Macaca fascicularis *have been elucidated and new insights obtained about the epitopic structure of the CD38 ECD. We hope that, by providing the reagents for analysis of CD38 in the cynomolgus macaque, this study may expedite our understanding of the role of CD38 in human disease.

## Methods

### Cynomolgus CD38 cDNA cloning

PBMC were isolated by Ficoll-Paque PLUS (Amersham Biosciences) centrifugation from whole blood obtained from a female cynomolgus macaque housed according to state regulations at the RBM (Ivrea, Italy). Cells were cultured in medium with 5 μg/ml PHA (Sigma-Aldrich) for 72 h, harvested and resuspended in TRIzol RNA extraction solution (Invitrogen). RT-PCR was carried out using the Titan One Tube RT-PCR system (Roche Diagnostics) with 150 ng total RNA and the gene-specific primers derived from the human CD38 sequence (GenBank accession no. D84278): forward 5'-AGT TTC AGA ACC CAG CCA-3' (corresponding to nt -84 to -66 upstream of the ATG initiation codon); reverse 5'-ATT GAC CTT ATT GTG GAG G-3' (corresponding to nt 102–121 downstream of the TGA stop codon). After RT for 50°C for 30 min, the cDNA was amplified by two rounds of PCR: 5 min at 94°C, followed by 30 (first round) or 35 (second round) cycles at 94°C for 30 s, 54°C for 30 s and 2 min at 72°C. The product was gel purified and cloned into the pGEM-T Easy vector (Promega). The inserts of three recombinant clones were analyzed by automated sequencing (Applied Biosystems).

### Analysis of simian genomic DNAs

Cynomolgus macaque (15 samples) and chimpanzee (5 samples) genomic DNAs were obtained as described [59]. The eight exons of the cynomolgus CD38 gene were amplified by PCR (45 cycles, annealing temp 52°C) using primers known to amplify human *CD38 *exons [41] while the *CD38 *promoter of cynomolgus macaque was amplified (15 cycles annealing at 52°C, followed by 35 cycles with 0.3°C touchdown) with a forward primer from the human *CD38 *promoter (5'-GAA GAG GCA AGA AAA GCC-3') and reverse primer chosen from macaque *CD38 *exon 1 (5'-AACTCG CAG TTG GCC ATA-3'). The chimpanzee CD38 promoter was amplified with the human CD38 sequences. The 5' end of cynomolgus *CD38 *intron 1 was amplified (same conditions used for exon amplification but 57°C annealing and 1.5 M MgCl_2_) with forward primer 5'-CCG TCC TGG CAC GAT GCG TCA AG-3' from macaque exon 1, and reverse primer 5'-ACA CCC TCC TCC CCT ACC ACA GG-3' taken from human *CD38 *intron 1. Amplicons were gel purified and analysed by automated sequencing. Alignments were performed with CLUSTALW (ExPASy, Swiss Institute of Bioinformatics).

### Cell lines and antibodies

NIH/3T3 murine fibroblast and COS-7 monkey kidney cell lines were from the ATCC. Production of SL-691 and SL-999 cynomolgus B lymphoblastoid cell lines was previously described [60]. The mutant human CD38^Δ285 ^plasmid [48] was kindly provided by Dr. Toshiaki Katada (University of Tokyo, Japan) while the MT2^Δ285 ^transfectant was kindly provided by Dr. Umberto Dianzani (A. Avogadro University of Eastern Piedmont, Novara, Italy) [29]. Cells were maintained in RPMI 1640 medium with 10% heat-inactivated FCS, penicillin/streptomycin. The murine anti-human CD38 mAbs AT1 (hybridoma kindly provided by Dr. Jo Hilgers, BioProbe AV, Amstelveen, The Netherlands), SUN-4B7, OKT10, IB4, IB6 and HB7 were produced in-house from hybridoma culture supernatants. CBT3G, a murine anti-human CD3 mAb, was used as IgG control. F(ab')_2 _goat anti-mouse Ig-FITC was used as secondary antibody (Jackson ImmunoResearch Laboratories).

### Expression of macaque and human CD38

cDNAs were cloned into pcDNA3.1/V5-His-TOPO expression vector (Invitrogen). Stable transfected cell lines were produced in NIH/3T3 by electroporation (250 V, 960 μF at 20°C), selected for 3–4 weeks in G418 after which isolated clones were picked and transferred to 96-well plates.

### Ecto-GDPR cyclase activity

Ecto-GDPR cyclase activity of intact cells was determined as previously described [29,30,61]. Briefly, parental and transfected NIH/3T3 were used at 2.5 or 5 × 10^5 ^cells/ml in PBS. For RBCs, 420 μl packed volume were brought to 1 ml by addition of NGT buffer (0.15 M NaCl, 5 mM glucose, 10 mM Tris. Cl, pH 7.4). To 1 ml cell suspensions 10 μl 10 mM NGD (Sigma) in 20 mM Tris, pH 7.4 or 10 μl buffer (control) were added. After 30 min at 37°C, supernatants were collected after brief centrifugation. Supernatants were analysed by fluorescence spectrometer set at excitation wavelength 300 nm and emission wavelength 410 nm. Cell lines were measured in triplicate in three independent experiments; RBCs from two different animals were tested once; RBCs from humans were tested in duplicate in two independent experiments.

### Western blotting

Cells were lysed in NP-40 buffer (150 mM NaCl, 1.0% NP-40, 50 mM Tris pH 8.0) containing protease inhibitors. Samples (20 μg protein/lane) were analysed by 8 or 10% SDS-PAGE and transferred to PVDF membranes (Bio-Rad Laboratories, Hercules, CA). Membranes were blocked in 5% milk/TBST, incubated for 2–3 h at RT with mAb supernatant with 1% milk, and incubated with horseradish peroxidase-labeled anti-mouse IgG (PerkinElmer) followed by ECL visualization.

### Production of anti-cynomolgus macaque CD38 mAbs

BALB/c mice (Charles River Laboratories) were anaesthetized with Avertin i.p. injection and immunized by intrasplenic injection with 300 μl containing 5 × 10^5 ^live NIH/mac38 cells in PBS. Eight days later, mice received an i.p. boost of NIH/mac38 cells, and mouse sera tested 8 days later. The spleen of one mouse that responded to immunization was selected for fusion to P3.X63.Ag8.653 murine myeloma cell line. Hybridoma supernatants were screened for reactivity with the immunizing cells and lack of reactivity with the parental cell line. Positive hybridomas were cloned by limiting dilution. MAbs were used in the form of supernatants containing NaN_3_.

### Analysis of surface CD38 expression

Surface expression of cynomolgus and human CD38 was determined by IF. Briefly, 2 × 10^5 ^cells/sample were incubated with 100 μl mAb supernatant/NaN_3 _for 1 h at 4°C, and 30 min at 4°C with FITC-labeled secondary Ab. Background fluorescence was established with control IgG antibody. Cells were analysed immediately either by fluorescence microscope, FACSCalibur flow cytometer (10,000 events acquired) with CellQuest software (Becton Dickinson) or Olympus FV3000 confocal microscope with Nomarski optics for differential interference contrast (DIC) and FluoView 300 software.

### Modulation of surface CD38 by treatment with DTT

Cells were detached with 1 mM EDTA/PBS, washed and resuspended in complete medium at a concentration of 2 × 10^6 ^cells/ml. One hundred μl 100 mM DTT stock was added per ml cell suspension, kept for 45 min in a 37°C incubator and washed twice with PBS/BSA/NaN_3 _before analysis.

## Authors' contributions

EF carried out cellular, biochemical and enzymatic analyses, devised the comparative epitope mapping and wrote the manuscript; MO cloned the cDNA and participated in the genomic DNA analyses; PV carried out mAb production and confocal microscopy, and participated in cellular and biochemical assays; EO carried out FACS analyses; SC participated in the genomic DNA analyses and contributed primate DNA samples; FS designed and participated in the cDNA and genomic DNA analyses; FT carried out expression analyses in macaques; FM designed and supervised mAb generation, and edited the manuscript. All authors read and approved the final manuscript.

**Table 3:** Inter-species cross-reactivity of anti-CD38 mAbs

	**Macaque CD38**		**Human CD38**	
**MAbs**	**native**	**DTT**	**native**	**DTT**
***Anti-macaque CD38***				
KK1B5	**+**	**-**	**+**	**-**
KK4E5	**+**	**+**	**-**	**-**
KK6A11	**+**	**+**	**-**	**-**
KK9H4	**+**	**-**	**+**	**-**
***Anti-human CD38***				
IB4	**-**	**-**	**+**	**+**
IB6	**-**	**-**	**+**	**+**
HB7	**-**	**-**	**+**	**+**
AT1	**+**	**-**	**+**	**-**
OKT10	**+**	**-**	**+**	**-**
SUN-4B7	**+**	**-**	**+**	**-**
